# Relationship Between Plasma Acid Sphingomyelinase and Alteration in Taste and Smell as Indicator of Long COVID in Pregnant Women

**DOI:** 10.3390/reports7040104

**Published:** 2024-11-21

**Authors:** Federico Fiorani, Giulia Moretti, Laura Cerquiglini, Chiara Gizzi, Giulia Gizzi, Paola Signorelli, Samuela Cataldi, Tommaso Beccari, Elisa Delvecchio, Claudia Mazzeschi, Stefania Troiani, Elisabetta Albi

**Affiliations:** 1 Department of Pharmaceutical Sciences, University of Perugia, 06126 Perugia, Italy; federico.fiorani@dottorandi.unipg.it (F.F.); samuela.cataldi@gmail.com (S.C.); tommaso.beccari@unipg.it (T.B.); 2 Department of Philosophy, Social Sciences and Education, University of Perugia, 06123 Perugia, Italy; giulia.moretti1@unipg.it (G.M.); giulia.gizzi@unipg.it (G.G.); elisa.delvecchio@unipg.it (E.D.); claudia.mazzeschi@unipg.it (C.M.); 3 Complex Structure of Neonatology and Neonatal Intensive Care, Azienda Ospedaliera Santa Maria della Misericordia, 06120 Perugia, Italy; laura.cerquiglini@ospedale.perugia.it (L.C.); stefania.troiani@unipg.it (S.T.); 4 Department of Medicine and Surgery, Section of General and Specialist Pediatrics, University of Perugia, 06120 Perugia, Italy; chiara.gizzi@studenti.unipg.it; 5 Department of Health Sciences, University of Milan, 20142 Milan, Italy; paola.signorelli@unimi.it; 6 Institute for Molecular and Translational Cardiology (IMTC), San Donato Milanese, 20097 Milan, Italy

**Keywords:** taste, smell, sphingomyelin, sphingomyelinase, pregnancy, long COVID

## Abstract

**Background:** Persistent alterations in taste and smell affect a significant proportion of individuals following COVID-19, representing a component of post-acute COVID-19 syndrome, commonly referred to as long COVID. The degradation of sphingomyelin by acid sphingomyelinase is regarded as a biomarker for acquired demyelinating neuropathies. **Objectives:** This study was aimed to enroll women who contracted COVID-19 during pregnancy and experienced persistent alterations in taste and/or smell for more than 1 year post-infection, in comparison to pregnant women without any disturbances in these senses. **Methods:** The patients were subjected to a questionnaire investigating smell and taste disorders more than 1 year after the infection. Then, the levels of acid sphingomyelinase in the plasma of the participants were assessed. **Results:** The results showed that in women who had been pregnant and who had been infected with SARS Cov-2 during the COVID period and who still had taste and smell disorders 1 year later, plasma acid sphingomyelinase levels were double that of pregnant women who had contracted the infection during the COVID period but had not reported taste and smell disorders and that of pregnant women analyzed after the COVID period. **Conclusions:** The results suggest a hypothesis that the persistence of sensory disturbances in long COVID was probably due to a failure to utilize brain circuitry with demyelination resulting from chemosensory dysfunction of the olfactory epithelium.

## 1. Introduction

Taste and smell are strictly regulated by the nervous system. The gustatory system is mediated by clusters of heterogeneous taste receptors cells (TRCs) organized as taste buds on the tongue which allow the perception of salty, sour, bitter, and sweet. The taste nerve pathway projects to the gustatory thalamus and then to the gustatory insula [1]. In the orbitofrontal cortex, there is a combination of gustatory input, olfactory input from the piriform cortex, and visual input from the temporal lobe [1]. Recently, attention has focused on identifying the molecular mechanisms that underlie taste by identifying specific proteins that act via G protein-coupled receptors (GPCR) and specific signal transmission pathways [2]. The structure and function of GPCRs are regulated by membrane lipids, such as cholesterol and sphingomyelin (SM) [3]. It is known that SM is essential for myelin sheath, a multilayered membrane structure that insulates axons, and plays a role in nerve impulse transmission, in presynaptic plasticity, and in neurotransmitter receptor localization [4]. In some neurodegenerative diseases, alteration of taste function correlates with myelin-related lesions in the central nervous system [5]. Elevated acid sphingomyelinase (aSMase) levels, by degrading SM, suggest disease onset or progression of neurological disorders, including major depression, ischemic stroke, amyotrophic lateral sclerosis, multiple sclerosis, and some neurodegenerative diseases such as Alzheimer’s disease [6]. Moreover, alteration or total loss of taste perception can be an early indication of infection, as occurred in the Severe Acute Respiratory Syndrome Coronavirus 2 (SARS-CoV-2) pandemic [7]. Currently, there are no data on the relationship between the alteration of taste and smell in subjects who have had the SARS-CoV-2 infection and aSMase. Here, we carried out a pilot study to identify aSMase as a marker of taste-smell dysfunction in long COVID in pregnant women of the Umbrian population. The choice of this limited sample is motivated by the fact that the loss of taste and smell aggravates the uncomfortable situation of pregnant women, limiting the use of desirable nutrition useful for the correct quality of future breastfeeding.

Clinical Significance: From the results it is clear that aSMase can be a marker used to evaluate sensory deficits after viral infection, even some time after infection. The test can be of great clinical use for diagnostic purposes and for following any therapy.

## 2. Material and Methods

### 2.1. Study of Population

Thirteen new mothers who were pregnant during the COVID-19 period between May 2021 and March 2022 at the gynecological clinic of the “Santa Maria della Misericordia Hospital in Perugia” were included in the study one year after their discharge from hospital. The inclusion criteria included: Italian language, informed consent signed, positive test for SARS-CoV-2 infection, and no other pathologies. To ensure a homogeneous population, the mothers included in the study had an average age of 34.31 ± 5.90 and a weight before the start of pregnancy of 62.87 ± 9.11. All had completed their pregnancy with a weight gain of 12.22 ± 2.78 and a newborn weight of 3342.31 ± 651.13. To test the effect on alterations in taste and smell as effects of long COVID, all mothers who had SARS-CoV-2 infection, during pregnancy, were included in the study one year after their discharge from hospital.

The exclusion criteria were mothers aged under 27 and over 40, with obesity or pathological thinness, with pregnancy weight gain of more than 15 kg and with other specific symptoms of long COVID such as dyspnea, chronic cough, fatigue, cognitive impairment, and psychological symptoms [8].

Ten women who were pregnant after May 2022, outside of the pandemic period in Italy, negative for SARS-CoV-2 infection, were considered as controls.

The project was approved by the Bioethics Committee of the University of Perugia (n.73095 del 1 March 2022) and all procedures were performed accordingly. Participation in the study was on a voluntary basis and the mothers signed informed consent for participation. Participants were anonymized and no sensitive data were collected.

### 2.2. Questionnaire

All the women participating in the study were given a questionnaire asking for information on age, weight before the start of pregnancy and weight after pregnancy, weight of the newborn, previous and/or current pathologies, infection with SARS-CoV-2, current presence of taste and/or smell disturbance

### 2.3. Materials

RNAqueous^®^-4PCR kit was from Ambion Inc. (Austin, TX, USA). TaqMan-Array 96-well plates were purchased from Applied Biosystems (Foster City, CA, USA). RNAqueous^®^-4PCR kit was from Ambion Inc. (Austin, TX, USA). SDS-PAGE molecular weight standards were purchased from Nzythech (Lisboa, Portugal).

### 2.4. Quanitative Real-Time RT-PCR

Patient serum was used for total RNA extraction using the RNAqueous-4PCR kit, as previously described [9]. Samples were treated with RNase-free DNase to prevent the amplification of any genomic DNA present. Samples were dissolved in RNase-free water and the amount of total RNA was quantified by measuring absorbance at 260 nm (A260). RNA purity was assessed by the A260/A280 ratio. The A260/A230 ratio was also used to indicate the presence of chemical contaminants in nucleic acids. The extracted RNA was immediately frozen and kept at −80 °C. Prior to cDNA synthesis, RNA integrity was assessed by 1.2% TAE agarose gel electrophoresis. The cDNA was synthetized using 1 μg of total RNA for all samples using the High-Capacity cDNA Reverse Transcription kit under the following conditions: 50 °C for 2 min, 95 °C for 10 min, 95 °C for 15 s, and 60 °C for 1 min, for a total of 40 cycles. The following target gene was analyzed: sphingomyelin phosphodiesterase 1 (*SMPD1*, Hs03679347^_^g1). (GAPDH, Hs999905^_^m1) and 18S rRNA (S18, Hs99999901^_^s1) were used as house-keeping genes. Relative mRNA expression levels were calculated as 2^−ΔΔCt^, comparing the results of treated samples with the control samples.

### 2.5. ELISA Kit

The protein concentration was measured as previously reported with modifications [10]. To test the content of aSMase, Human Acid Sphingomyelinase SimpleStep Elisa kit from Abcam (Cambridge, UK) was used.

### 2.6. Statistical Analysis

A *t*-test (* *p* < 0.05) was used to evaluate the differences in gene expression and protein content of aSMase between women who had COVID-19 during pregnancy and who, one year later, still had a taste and/or smell disorder; and women who had COVID-19 during pregnancy; and who had a transient taste and/or smell disorder and therefore had no longer had the disorder after one year.

## 3. Results and Discussion

Since the gene expression and protein content of serum aSMase are very delicate tests that are affected by the influence of various factors such as age, weight, eating habits, etc., a very homogeneous population of patients was chosen (see “Study of population”, n° 13 participants). In addition, women who had COVID-19 during pregnancy (not the general population) were chosen to consider how a lack of taste and smell can make the experience of pregnancy in the COVID-19 period even more difficult. The results showed that eight women (61%) who had COVID-19 during pregnancy still had taste and/or smell disorders one year later, of which two women (15%) presented only taste disorder and one woman (7.7%) presented only smell disorder. Five women (38%) had no alterations in taste or smell. As controls (CTR), 10 women were included in the study.

**Figure 1:**
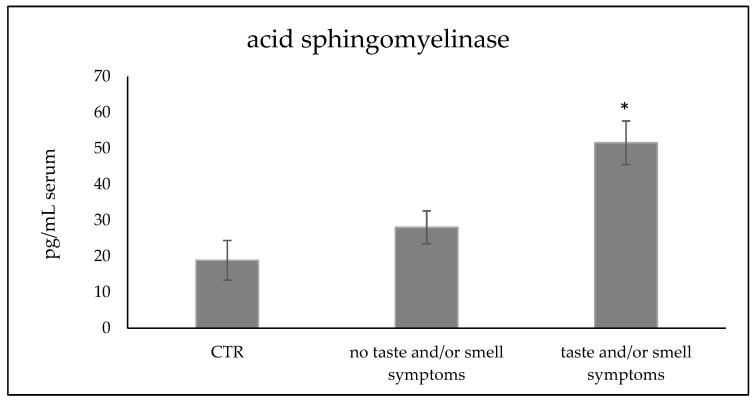
aSMase level in the serum of women who had infection of SARS-CoV-2 in pregnancy one year before the analysis. Comparison among women controls (CTR, 10 mothers), women who had alterations in taste and/or smell (eight mothers) and women without alterations (five mothers). Data were expressed as the mean ± SD of three independent experiments performed in triplicate for each patient. * *p* < 0.05 women with disturbance in taste and smell versus women without disturbance in taste and smell and versus CTR.

Interestingly, one year after SARS-CoV-2 infection, mothers with taste and smell disturbances had a higher serum aSMase level (51.54 ± 6.07 pg/mL) than those without taste and smell alterations (28.05 ± 4.57 pg/mL, Figure 1). As expected, the values of the mothers who had no alterations in taste and smell did not differ significantly from those of control mothers (18.87 + 5.5 pg/mL) (Figure 1). Whether, in the group of mothers who had smell and/or taste disorders, the two mothers who only had smell disorder were considered, the aSMase value was 48.89 + 4.21 pg/mL; the only mother who only had a taste disorder had an aSMase value of 62.44 + 4.31 pg/mL. All analyses were performed in triplicate for each mother.

**Figure 2:**
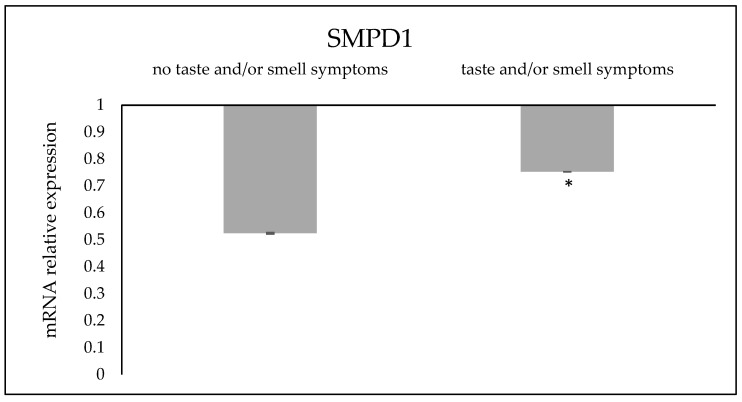
Expression of SMPD1 gene in serum of women who had infection of SARS-CoV-2 in pregnancy one year before the analysis. GAPDH and 18S rRNA were used as housekeeping genes. mRNA relative expression levels were calculated as 2^−ΔΔCt^, comparing the results of the COVID-19 patients with the control sample (CTR, without COVID-19) equal to one, the origin of the axes. Data are expressed as the mean ± SD of three independent experiments performed in duplicate for each patient. * *p* < 0.05 versus CTR.

The change in protein content did not correspond to changes in expression of the RNA coding for aSMase (Figure 2). In fact, the expression of the SMPD1 gene was low in both mothers without or with alterations in taste and/or smell in comparison with control samples.

The results suggested that there was probably a defect in the catabolism of aSMase which therefore accumulated and continued to degrade the SM, altering the structure of the gustatory and olfactory system cells. As reported above, in some neurodegenerative diseases alteration of taste function correlated with myelin-related lesions in the central nervous system [5]. Experimentally, demyelination of the white matter of the olfactory system (lateral olfactory tract and anterior commissure) was induced from the administration of lysolecithin [11] that acted on membrane lipids and stimulated endogenous lysolecithin [12]. It is possible that the production of endogenous lysolecithin following viral infection could induce activation of aSMase with degradation of myelin. Khodanovich et al. have reported research on post-COVID-19 neurological disorders focusing attention on demyelination which could be the consequence of the inflammation or direct effect of the virus on the oligodendrocytes with consequent myelin damage [13]. The authors, however, reported the scarcity of supporting clinical articles, probably due to the use of conventional MRI, underlining the need for more specific and innovative research. Tsukahara et al. stated that the lesion responsible for the olfactory alteration in COVID-19 was not direct but indirect as it did not concern the neurons but the support cells in the olfactory epithelium [14]. In support, Butowt et al. hypothesized that the basis of the loss of smell post-COVID-19 was a death of infected supporting cells in the olfactory epithelium with consequent alteration of the mucus and retraction of the cilia on the olfactory receptor neurons with alteration of the olfactory signal transduction within the olfactory cilia [15]. Epithelial regeneration could explain the rapid recovery that many patients had. It is possible to hypothesize that both mechanisms, direct and indirect, are involved in determining taste and smell disorders post-COVID-19 in different ways with short- and long-term effects.

Our results suggested that the increase in aSMase was correlated with taste and/or smell disturbance as an effect of long COVID. The possibility that other nerves were involved cannot be ruled out. For this study, only women who exclusively had alterations in taste and smell were chosen. In the present study, the persistence of the taste and/or smell disorder one year after the SARS-CoV-2 infection was reported by the mothers included in the study, responding to a targeted questionnaire. In recent reviews investigating taste disorder during COVID-19, it was shown that during the disease a high percentage of subjects reported a lack of taste but, following evaluated psychophysical taste tests, the percentage was strongly reduced [16,17]. It is possible that if the study participants had been subjected to the same tests, the percentages reported in the present study could also have been lower. In any case, participants were subjects who recognized the disorder because it had persisted for one year. Furthermore, all mothers who reported taste and/or smell disturbances had higher aSMase values than mothers who did not have such disturbances.

## 4. Conclusions

The present study represents the first observation on the relationship between serum aSMase level and taste and smell disturbance one year after SARS-CoV-2 infection. Data suggest that serum aSMase may be a marker of long COVID.

## Data Availability

All the data generated in this study has been included in this manuscript, further inquiries can be directed to the corresponding author.
